# Insights into gene expression profiling of natural resistance to coccidiosis in contrasting chicken lines

**DOI:** 10.1186/1753-6561-5-S4-S26

**Published:** 2011-06-03

**Authors:** Thomas Heams, Bertrand Bed'hom, Emmanuelle Rebours, Florence Jaffrezic, Marie-Hélène Pinard-van der Laan

**Affiliations:** 1 INRA/AgroParisTech, UMR1313 Génétique Animale et Biologie Intégrative, F-78352 Jouy-en-Josas Cedex, France; 2 INRA, PICT-Gem, F-78352 Jouy-en-Josas Cedex, France

## Abstract

Coccidiosis is a parasitic disease with major economic impact, one of whose main causative agents is *Eimeria tenella*. Chicken breeds display variable natural resistance to this disease. Unravelling the genetic bases of such variations could provide new clues for protection strategies. Transcriptomic experiments were conducted comparing resistant (Fayoumi) and susceptible (Leghorn) lines. Caecum and caecal tonsils were analysed. A global increase in differential gene expression following infection was observed for caecum comparisons, whereas a global decrease following infection was observed for caecal tonsils.

Gene lists for infected tissues display 40 genes in common across breeds, 20 of which were specific to infected tissues. Among these specific genes, 9 belong to the 100 more differentially expressed genes of the infected caecum comparison. Gene expression networks were constructed in parallel, identifying highly connected genes. Comparing information from differential gene lists and gene network analysis allows one to highlight potential pivotal genes in the infection process, one of which was located in a putative significant QTL region for infection associated lesions.

## Background

Coccidiosis is a disease with major global impact on several animal breeding species. It is one of the major diseases impacting poultry production with estimated of world losses averaging 1 billion $ / Year [1]. One of the main causative agents is the protozoan *Eimeria tenella*, an internal obligatory parasite, among other *Eimeria* species. During its life cycle, free stages invading epithelial cells alternate with a highly resistant stage (oocyst) adapted to long term environmental survival. The caecum is the primary site of infection. When infected, chickens (*Gallus gallus*) present bloody droppings, stop feeding, face dehydration and huddle together. High mortality is mainly due to excessive blood losses, with recovering individuals likely to develop chronic illness.

Several therapeutic strategies are currently in use. Anticoccidial drugs exist but cases of drug resistance are developing, and their use is evermore prohibited by regulations. Vaccination is partly possible, but it displays limitations due to multiple *Eimeria* species. Good farming practices avoiding excessively dense animal concentrations are highly recommended to this extent. Therefore, an improved knowledge of natural genetic resistance can pave the way towards protection against coccidiosis. Remarkably, chicken lines naturally display various degrees of resistance to *E. tenella* infection: the Egyptian Fayoumi line is known to be highly resistant compared to common layer breeds such as the White Leghorn [2]. Recent findings have located QTL regions related to lesions associated with this infection [3]. Here, we present a complementary approach using transcriptomic methods, which is the first to our knowledge to describe the effects of *E. tenella* infection on these lines. These results strengthen and broaden previous results from one of the above mentioned QTL studies.

## Methods

### Experimental design

Two tissues were studied: caecum and caecal tonsils (a lymphoid organ associated with caecum). For each tissue we first compared four to five Fayoumi (resistant line) and five White Leghorn (susceptible line) individuals that did not undergo infection, as control experiments. Then we performed similar comparisons for infected individuals at day 6 post infection. This design allowed us to make direct comparisons between lines, as well as between tissues and between infection status, and to discriminate between these different effects.

### Microarrays

These transcriptomic experiments were performed using microarrays. Briefly, mRNA samples were directly extracted with Qiagen RNeasy Kit from disrupted tissues, including a DNAse step. mRNAs were checked for purity, quality and quantity prior to any further steps. Dye-swap comparisons were performed on chicken 20.6 k oligo microarrays. Microarrays were analysed with an Agilent G2565BA Scanner at 10 µm resolution. Signal quantification was performed with the Genepix pro 6.0 software.

### List of differentially expressed genes

Data analysis was performed using the Bioconductor Limma package. Raw results were normalised as usual to overcome any cy5/cy3 fluorescence bias. Normalization was performed with the "normalizeWithinArrays" function with a loess correction for the dye effect and no background correction. No further normalization was required across arrays. The dye swap design implied technical replicates. Correlation structure between these technical replicates was accounted for using the "duplicateCorrelation" option.

### Gene networks

Gene network reconstruction was based on Graphical Gaussian models and performed using the GeneNet R package [4].

## Results

### Global profiling of differential expression

In caecums, at a 5% Benjamini-Hochberg threshold, 1473 genes were found to be differentially expressed between the two infected lines, and 174 genes were differentially expressed between the two non-infected control lines. 42 of these genes were overlapping (present in both comparisons).

In caecal tonsils, at a 5% Benjamini-Hochberg threshold, 61 genes were found to be differentially expressed between the two infected lines, and 113 genes were differentially expressed between the two non-infected control lines. 17 of these genes were overlapping.

Thus, the two tissues display a consistent remodelling of their differential gene expression during infection, as opposed to the basal differential gene expression which is of a similar magnitude (174 and 113 genes) in the two tissues.

However, the two responses display apparently opposite trends: a global increase of differentially expressed genes is observed (from 174 to 1473 genes) in caecums, whereas a global decrease is observed (from 113 to 61 genes) in caecal tonsils. This might be related to the fact that the two tissues are not impacted at the same time of the infection process, and will be further investigated. But due to the quite low overlap of genes between infected and control experiments, the main and common feature of these infection effects remains the global remodelling of differentially expressed gene list.

In order to narrow the core group of genes globally involved in the infection process, we compared the different gene lists. 40 genes were found common to both tissues’ “infected” comparisons. 20 of these genes were only found in both “infected” comparison lists (that is to say not in the “control” lists), and therefore can be labelled as “specific”. Among these specific genes, 9 belonged to the 100 most differentially expressed genes of the “infected” caecum comparison, and therefore can be labelled as “top specific”.

### First insights in gene network analysis

**Figure 1:**
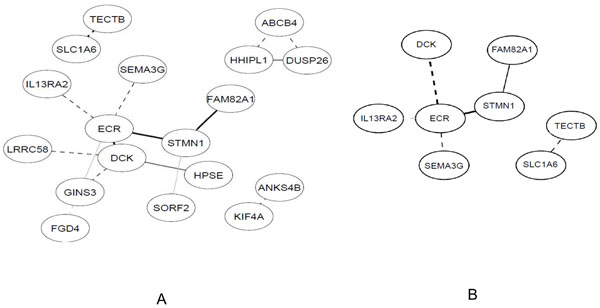
**Gene Expression networks of the caecum infection by *E. tenella.*** (A) Significant edges at 20% FDR (B) Significant edges at 10% FDR. Plain lines: positive relationship; dashed lines: negative relationship

Networks were obtained using the 100 most differentially expressed genes between the two infected lines for the caecum. Significant edges were found at a 10% and 20% local false discovery rate (FDR) threshold. The corresponding networks (displaying respectively 8 and 18 genes) are presented in figure 1.

### Comparing gene interaction network and differential expression information

4 of the above described 9 top specific genes were found in the 20% network, and their corresponding proteins are: HPSE (GGA4 - heparan cliving protein involved in leucocytes migration pathway), SORF2 (GGA18 - maps in a highly significant QTL region for lesion [3]), ABCB4 (GGA2 - defence against xenobiotics), DUSP26 (GGA22 - signalling pathway modulation). In addition, 2 of the 40 common genes were found in the 10% network: TECTB (GGA6 - matrix protein) and an ECR (GGA2- evolutionary conserved region/no gene annotation– similar to a replication licensing factor).

## Conclusions

These first results show the usefulness of performing and integrating different transcriptomic methods when studying the genetic bases of natural resistance to diseases. Microarrays allowed a global view of transcriptomic modification upon infection, detecting different trends and amplitude of gene expression changes in the two tissues. As a starting point, we focused on the common genes differentially expressed in both tissues. A detailed analysis of the differentially expressed genes lists in each tissue, including a clustering based on variation profiles, is currently ongoing. Comparing this information, enhanced by a two-tissue comparison, with gene network analysis obtained with the same list allowed the identification of core genes significantly associated with the day 6 post infection molecular phenotype. It is noteworthy that several of these genes are related to extracellular matrix (hereby not excluding leucocyte migration pathways associated with infection), and one of them, SORF2, maps into a previously described highly significant QTL region for lesions. This can be seen as a partial validation of these previous results, and demonstrates that combined QTL/transcriptome studies can lead to identification of candidate genes. Nonetheless, further refinements of these results are required, in order to better understand the biological meaning of the obtained networks. The status of highly differentially expressed genes not found in the gene networks will need special further investigation. In parallel, an extensive comparison with the above mentioned QTL data will help to understand why some regions are congruent in the two types of studies, and others not. In depth analysis of the gene network relationships will bring a more subtle view of the genome dynamics correlated with resistance or susceptibility to coccidiosis. Transcriptomics of intermediary steps of the infection kinetics will refine these initial results, provided that mRNA expression can be observed preceding clinical signs of infection such as lesions. Our results describe infection by *Eimeria tenella*, however other *Eimeria* species are alternative causative agents of coccidiosis. Comparing and interpreting similar results obtained with these different species might help decipher the specific and common features of coccidiosis infection at the molecular level.

These first results demonstrate the efficiency of a microarray strategy to identify genes and networks involved in infection. Further, they underline the usefulness of local breeds as genetic resources.

## Competing interests

The authors declare that they have no competing interests.

## Authors' contributions

TH co-designed the study, wrote the paper, performed all steps from RNA extraction from tissues to microarray hybridization, participated in the interpretation of the results. BB contributed to functional aspects of the study, collected samples, participated in the interpretation of the results and identified genes within networks. ER performed transcriptomic steps including image analysis and signal quantification. FJ co-designed the study, and performed the statistical and the gene network analyses. MHPvdL supervised and co-designed the study, performed the infection steps, and collected samples. All authors read and approved the final manuscript.
